# A comparison of multiple testing adjustment methods with block-correlation positively-dependent tests

**DOI:** 10.1371/journal.pone.0176124

**Published:** 2017-04-28

**Authors:** John R. Stevens, Abdullah Al Masud, Anvar Suyundikov

**Affiliations:** 1 Department of Mathematics and Statistics, Utah State University, 3900 Old Main Hill, Logan, UT 84322-3900, United States of America; 2 Department of Biostatistics, Indiana University Fairbanks School of Public Health and Indiana University School of Medicine, Indianapolis, IN 46202, United States of America; 3 BioStat Solutions, Inc., 5280 Corporate Drive, Suite C200, Frederick, MD 21703, United States of America; National Institute of Environmental Health Sciences, UNITED STATES

## Abstract

In high dimensional data analysis (such as gene expression, spatial epidemiology, or brain imaging studies), we often test thousands or more hypotheses simultaneously. As the number of tests increases, the chance of observing some statistically significant tests is very high even when all null hypotheses are true. Consequently, we could reach incorrect conclusions regarding the hypotheses. Researchers frequently use multiplicity adjustment methods to control type I error rates—primarily the family-wise error rate (FWER) or the false discovery rate (FDR)—while still desiring high statistical power. In practice, such studies may have dependent test statistics (or p-values) as tests can be dependent on each other. However, some commonly-used multiplicity adjustment methods assume independent tests. We perform a simulation study comparing several of the most common adjustment methods involved in multiple hypothesis testing, under varying degrees of block-correlation positive dependence among tests.

## Introduction

A common initial question in a genomic study is to identify genes whose expression levels change with the different levels of some variable of interest such as a covariate or response variable. The response variable could be a clinical outcome or survival time, whereas the covariate could be the dose of a drug, time, treatment/control group, and so forth [1]. Questions in spatial epidemiology can involve identifying locations where disease risk is associated with an environmental variable [2]. Brain imaging studies can involve identifying voxels (essentially very specific brain regions) that exhibit different levels of brain activity in response to some stimulus [3, 4]. These three fields (genomics, spatial epidemiology, and brain imaging), among many other fields, all can involve situations where potentially thousands (or more) features (genes, locations, voxels) are tested for differential abundance (expression, risk, brain activity) between levels of some variable of interest.

Multiple hypothesis testing is often applied to identify differentially abundant features across different levels of the variable of interest. The null hypothesis for each feature is that the abundance levels are not associated with the variable of interest. With thousands (or more) of null hypotheses to test, it becomes important to control the overall type I error rate at level *α* while maintaining the desired statistical power (= 1—type II error rate). Because of the mixture of true and false null hypotheses, the obtained p-values follow different types of distributions, for example Beta distributions instead of the Uniform distribution (which would result if all null hypotheses were true). Multiple comparison adjustment methods can control the FWER, FDR [5], or positive false discovery rate (pFDR) [6]. In higher-dimensional studies, most often controlling the FDR, or the pFDR, ensures more statistical power than controlling the FWER [1].

Some commonly-used multiple testing adjustment methods (such as the original FDR method by Benjamini and Hochberg (1995) [5]) assume independence of tests, which in gene expression studies translates to a questionable assumption that all genes operate independently. (Corresponding and similarly questionable assumptions in other fields would be the independence of spatial locations, or the independence of different regions of the brain.) Other multiple testing adjustment methods claim to provide error rate control under certain (or even arbitrary) dependence types among test results [7, 8]. It would be useful to know how various adjustment methods perform under various levels of test dependence. The objective of this paper is to make such an evaluation so that a methodological recommendation can be made, leading to better-justified conclusions from high-dimensional data analysis. The paper is arranged in the following manner: first, in the “Methods: controlling the error rates” Section (and greater detail in Section A of S1 File) we summarize several procedures in the literature to control error rates in multiple comparisons. Then in the “Methods: simulation analysis” Section we propose a simulation framework and analyze several multiple comparison procedures using simulation data sets. Finally in the “Results and Discussion” Section, we finish with some observations and recommendations.

## Methods: Controlling the error rates

Many multiple testing adjustment methods exist for controlling error rates. For our current purposes, we focus on several that are most commonly used and widely available to applied researchers. To ensure the main body of this article focuses on our novel contributions, we have summarized in Section A of S1 File the background literature on these methods, as well as a brief discussion on dependence structures. For control of the FWER, we consider the Bonferroni procedure [9], Šidák’s single step and step-down procedures [10, 11], the Holm procedure [12], the Hommel procedure [13], and the Hochberg procedure [14, 15]. For control of the FDR, we consider the Benjamini and Hochberg procedure [5], the Benjamini and Yekutieli procedure [7], the adaptive Benjamini and Hochberg procedure [16], the two stage Benjamini and Hochberg procedure [17], the q-value method [6], and the principal factor approximation method [8]. The multiple comparison procedures discussed in Section A of S1 File are shown in Table 1. These procedures are used to adjust p-values in our simulation analysis in the “Methods: simulation analysis” Section and to visualize results in the “Results and Discussion” Section.

**Table 1:** Abbreviations of multiple comparison procedures (and their corresponding controlled error rate), used in the text and in summary figures.

Procedure	Abbreviation	Error Rate
Bonferroni procedure	Bonferroni	FWER
Šidák single step procedure	Sidak SS	FWER
Šidák step down procedure	Sidak SD	FWER
Holm procedure	Holm	FWER
Hommel procedure	Hommel	FWER
Hochberg procedure	Hochberg	FWER
Benjamini and Hochberg procedure	BH	FDR
Benjamini and Yekutieli procedure	BY	FDR
Adaptive Benjamini and Hochberg procedure	ABH	FDR
Two stage Benjamini and Hochberg procedure	TSBH	FDR
q-value method	q-value	FDR
Principal factor approximation	PFA	FDR

## Methods: Simulation analysis

In this section we evaluate the performance of the multiple testing procedures from the “Methods: controlling the error rates” Section (and Section A of S1 File), under various dependence scenarios using simulated data sets. We simulated *m* test statistics (corresponding to *m* features in a hypothetical study) as $\underset{∼}{Z}∼N\left(\underset{∼}{\mu },\underset{∼}{\Sigma }\right)$. Here $\underset{∼}{\mu }$ is a length *m* vector of the expected differences for each test; in the high-dimensional study context, *μ*_*i*_ is the true magnitude of differential abundance for feature *i*. We considered two levels of *m*: 2000 (where the control of the FDR would generally be more meaningful) and 100 (where control of the FWER would generally be more meaningful). Our scenario of dependent test statistics (and subsequent p-values) is represented in the covariance matrix ($\underset{∼}{\Sigma }$), where we considered different numbers of correlated tests (or features) and varying levels of correlation. In addition, we considered different sizes of *μ*_*i*_ in order to compare the performance of these multiple comparison procedures. Our general hypothesis is constructed as follows:

$$\begin{array}{c} H_{i}^{0}:\mu_{i}=0\;\text{vs}\;H_{i}^{1}:\mu_{i}\neq 0\;\text{for}\;i=1,2,\ldots ,m. \end{array}$$

In our study the first and the most important dependency scenarios are represented in the covariance matrix (${\underset{∼}{\Sigma }}_{m\times m}$) of test statistics ($\underset{∼}{Z}$). Thus, the construction of $\underset{∼}{\Sigma }$ addresses two issues: (1) number of total correlated $\underset{∼}{Z}$ (corresponding to features) and (2) correlation value (*ρ*) of dependent $\underset{∼}{Z}$. Regarding the first issue, our main motivation is to examine the performance of various multiple comparison procedures when increasing the total number of correlated $\underset{∼}{Z}$. We considered two different total numbers of correlated $\underset{∼}{Z}$: 120 and 360 (out of 2000 total) for FDR control; 18 and 36 (out of 100 total) for FWER control. We also considered these dependent test statistics in blocks in order to distribute the number of dependent $\underset{∼}{Z}$ into six disjoint but equal-sized sets of correlated tests. For example, in the case of 120 dependent tests, we have six blocks, each with twenty dependent $\underset{∼}{Z}$. In each case, the dependent $\underset{∼}{Z}$ of the first three blocks were always associated with the alternative hypothesis being true (*μ*_*i*_ ≠ 0), while the dependent $\underset{∼}{Z}$ of the remaining three blocks were associated with the null hypothesis being true (*μ*_*i*_ = 0). Then, we set all remaining test statistics to be independent. Therefore, the diagonal elements of the entire ${\underset{∼}{\Sigma }}_{m\times m}$ matrix consist of six blocks with the remaining diagonal elements being ones, and the off-diagonal elements being zeros. So all blocks under a specific total number of correlated $\underset{∼}{Z}$ always appear at diagonal positions of the entire $\underset{∼}{\Sigma }$ matrix. Indeed, each block is a symmetric matrix inside the $\underset{∼}{\Sigma }$ matrix.

As off-diagonal elements of the blocks on the diagonal of $\underset{∼}{\Sigma }$, we considered correlation coefficient values *ρ* ∈ {0, .2, .4, .6, .8, .99}. The values of *ρ* are chosen to represent a reasonable range of values. Non-negative *ρ* ensures that the covariance among $\underset{∼}{Z}$ is always non-negative. Thus, with such Gaussian $\underset{∼}{Z}$ with positive correlation, we satisfy the condition of positive regression dependency [18] (see “Dependence among test results: PRDS and MTP_2_” in Section A of S1 File). These correlation values measure how much the dependent $\underset{∼}{Z}$ are correlated with each other. Specifically, *ρ* = 0 indicates features are completely independent; in contrast, *ρ* = 0.99 indicates that the linear association between features’ test statistics is almost exact. Considering this block-correlation dependence structure, it helps to compare the performance of various multiple comparison procedures under the same degree of dependency in the same number of both true null and false null hypotheses. By changing the *ρ* values we obtain different off-diagonal elements in the blocks. Thus, we summarize the construction of our general ${\underset{∼}{\Sigma }}_{m\times m}$ matrix in such a way so that, under a given number of correlated $\underset{∼}{Z}$, all blocks (or symmetrical sub-matrices) appear along the diagonal of the $\underset{∼}{\Sigma }$ matrix, and the off-diagonal elements of the blocks preserve the degree of dependency of correlated $\underset{∼}{Z}$. The following is the general form of our ${\underset{∼}{\Sigma }}_{m\times m}$ matrix:

$$\begin{array}{c} \underset{∼}{\Sigma }=\left[\begin{array}{c} \begin{array}{ccccc} \left[\begin{array}{cc} 1 & \rho \\ \rho & 1 \end{array}\right] & 0 & 0 & \cdots & 0\\ 0 & \left[\begin{array}{cc} 1 & \rho \\ \rho & 1 \end{array}\right] & 0 & \cdots & 0\\ 0 & 0 & \left[\begin{array}{cc} 1 & \rho \\ \rho & 1 \end{array}\right] & \cdots & 0\\ \cdots & \cdots & \cdots & \cdots & \cdots \\ 0 & 0 & 0 & \cdots & 1 \end{array} \end{array}\right] \end{array}$$ (1)

This block-diagonal structure of the $\underset{∼}{\Sigma }$ matrix affects the construction of our mean vector $\underset{∼}{\mu }$ for our $\underset{∼}{Z}$. For tests with a true null hypothesis, *μ*_*i*_ = 0, while for tests with a false null hypothesis, we set *μ*_*i*_ = *A* for some *A* > 0. For demonstration purposes, we use the same *A* for all false null hypotheses, allowing an inspection of the effect of *A*, and consider separately *A* ∈ {0.5, 1, 2, 3, 4, 5}. These values of *A* are chosen to represent a reasonable range of values.

Three of the six dependent blocks in the $\underset{∼}{\Sigma }$ matrix correspond to true null hypotheses (so their corresponding *μ*_*i*_ = 0), and the remaining three dependent blocks correspond to false null hypotheses (so their corresponding *μ*_*i*_ = *A*). In our simulations to consider FDR control, we considered 200 false null hypotheses (out of 2000 total hypotheses), thus we have either 140 (= 200 − 3 × 20) or 20 (= 200 − 3 × 60) completely independent tests with false null hypotheses, depending on the dependence group size. In simulations to consider FWER control, we considered 20 false null hypotheses (out of 100 total hypotheses), with either 11 (= 20 − 3 × 3) or 2 (= 20 − 3 × 6) completely independent tests with false null hypotheses, depending on the dependent group size. Notice that implicit in our simulation is the assumption that a group (or block) of dependent hypotheses will have a shared truth (nulls all true or all false). This assumption is made for computational convenience and to facilitate interpretation. The following is the general form of our ${\underset{∼}{\mu }}_{1\times m}$ vector:

$$\begin{array}{c} \mu =\left[\begin{array}{c} \begin{array}{ccccccccc} A & A & \cdots & A & 0 & 0 & 0 & \cdots & A \end{array} \end{array}\right] \end{array}$$ (2)

For any total number of dependent $\underset{∼}{Z}$ and for each simulation, we simulated *m* $\underset{∼}{Z}$ under a specific *A* and *ρ* combination. We performed our simulation 1,000 times considering a given number of total dependent $\underset{∼}{Z}$. Thus for each combination of ${\underset{∼}{\mu }}_{1\times m}$ vector and ${\underset{∼}{\Sigma }}_{m\times m}$ matrix, we generated 1,000 sets of *m* p-values. Next, we adjusted these p-values with the multiple comparison methods listed in the “Methods: controlling the error rates” Section (and Table 1) above to control the FDR (when *m* = 2000) or FWER (when *m* = 100) at *α* = 0.05. Finally, we estimated power, FDR, and FWER of the corresponding multiple comparison procedures by averaging for each procedure across simulations for each combination of *ρ*, *A*, and specific total number of dependent $\underset{∼}{Z}$. Because there is, of course, chance variability across simulations, we also obtain the standard deviations (across simulations) of the power, FDR, and FWER, allowing construction of approximate 95% confidence intervals of the true power, FDR, and FWER of each method by considering the average ± 2 SEM, where SEM is the standard error of the mean.

It is important to keep in mind the limitations and intent of this simulation. In practice, when features are dependent, it will not necessarily be with the same constant correlation in each dependent group, as represented in Eq 1. Similarly, in practice, when features are differentially abundant, they will not all be differentially abundant with the same magnitude (or even direction), as represented in Eq 2. Instead, there will be something of a mixture—dependent groups with varying strengths of dependence, and differentially abundant features with varying magnitudes (and direction) of change. However, the block correlation structure (which is a standard exploratory initial tool) and the differential abundance framework used in this simulation are not intended to fully recreate a complex biological system. Rather, the simulation is intended to give some insight into how the various multiplicity adjustment methods will perform on various components of this mixture—particularly as the strength of dependence (even if narrowly defined within this block correlation framework) and magnitude of differential abundance vary.

Finally, in this simulation the proportion of differentially abundant features is held constant at 10% (arbitrarily a low percentage), and the total number of features is held constant. When considering the FDR, we use *m* = 2000 features—arbitrarily a high number, which involves substantial-but-manageable computational expense in dealing with $\underset{∼}{\Sigma }$, and which is close to the number of features in a microRNA study [19, 20]. When considering the FWER, we use *m* = 100 features, arbitrarily a low number, but one which is reasonable in a pharmacogenomics PGx subgroup analysis [21] or methylation quantitative trait loci study [22]. We fix the percent differential abundance and number of features thus in the simulation, not because in practice we could assume the same percentage differentially abundant or the same number of features in all studies, but rather because our focus is on how the degree of dependence and magnitude of differential abundance (and not percentage differentially abundant or number of features) affect the performance of the multiplicity adjustment methods considered here. Accordingly, rather than varying all simulation characteristics (such as percentage differentially abundant, or the total number of features), we instead vary the simulation characteristics of greatest interest for our purposes (degree of dependence and magnitude of differential abundance).

## Results and discussion

**Fig 1:**
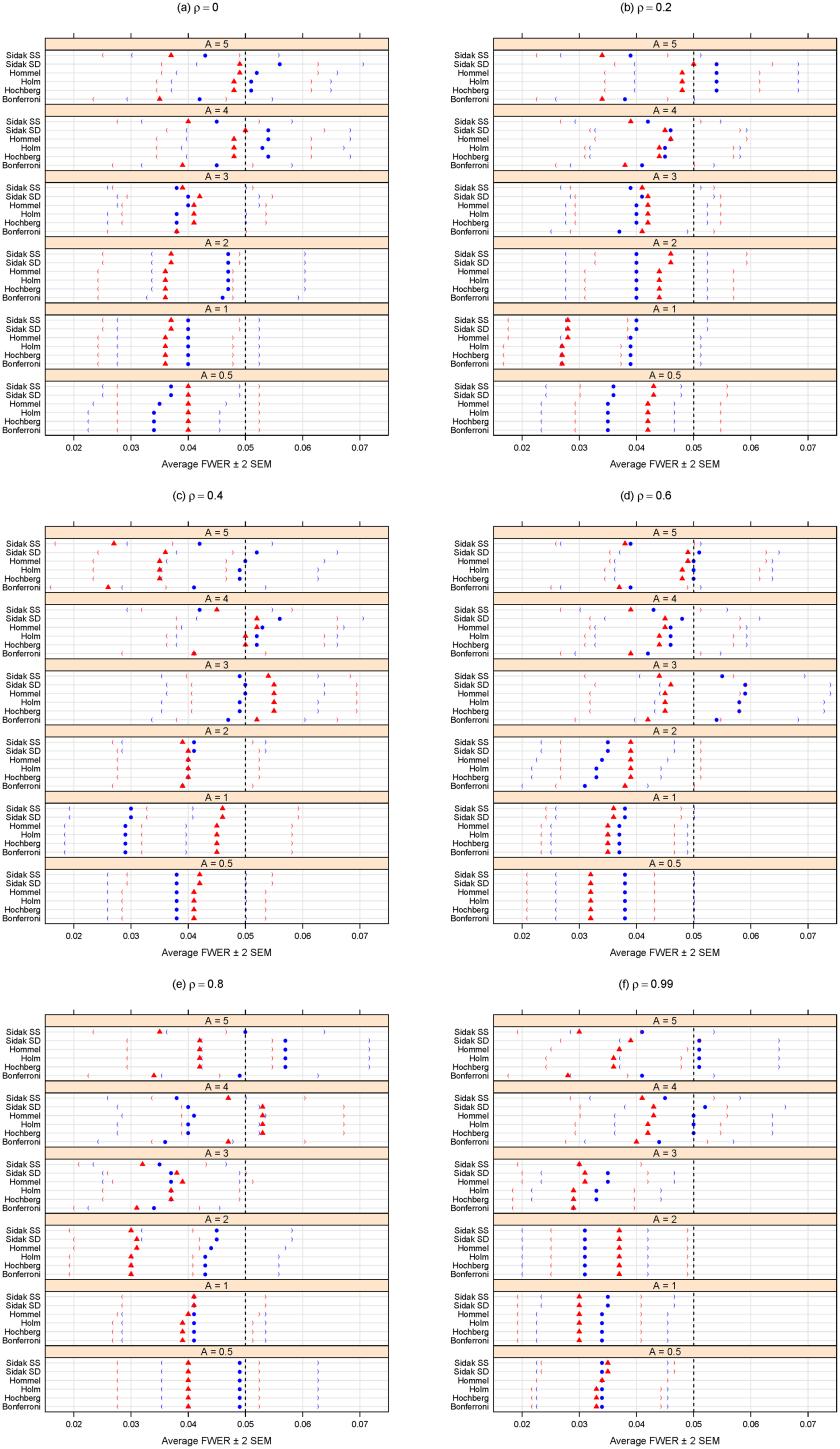
Average FWER for different methods purporting to control the FWER at *α* = 0.05. *A* can be thought of as the magnitude of differential abundance for truly differentially abundant features, and *ρ* is the true correlation within blocks of dependent tests. The blue solid circles represent the case of 18 dependent tests (out of 100 total), whereas the red solid triangles are for the case of 36 dependent tests. Parentheses indicate ± 2 SEM.

**Fig 2:**
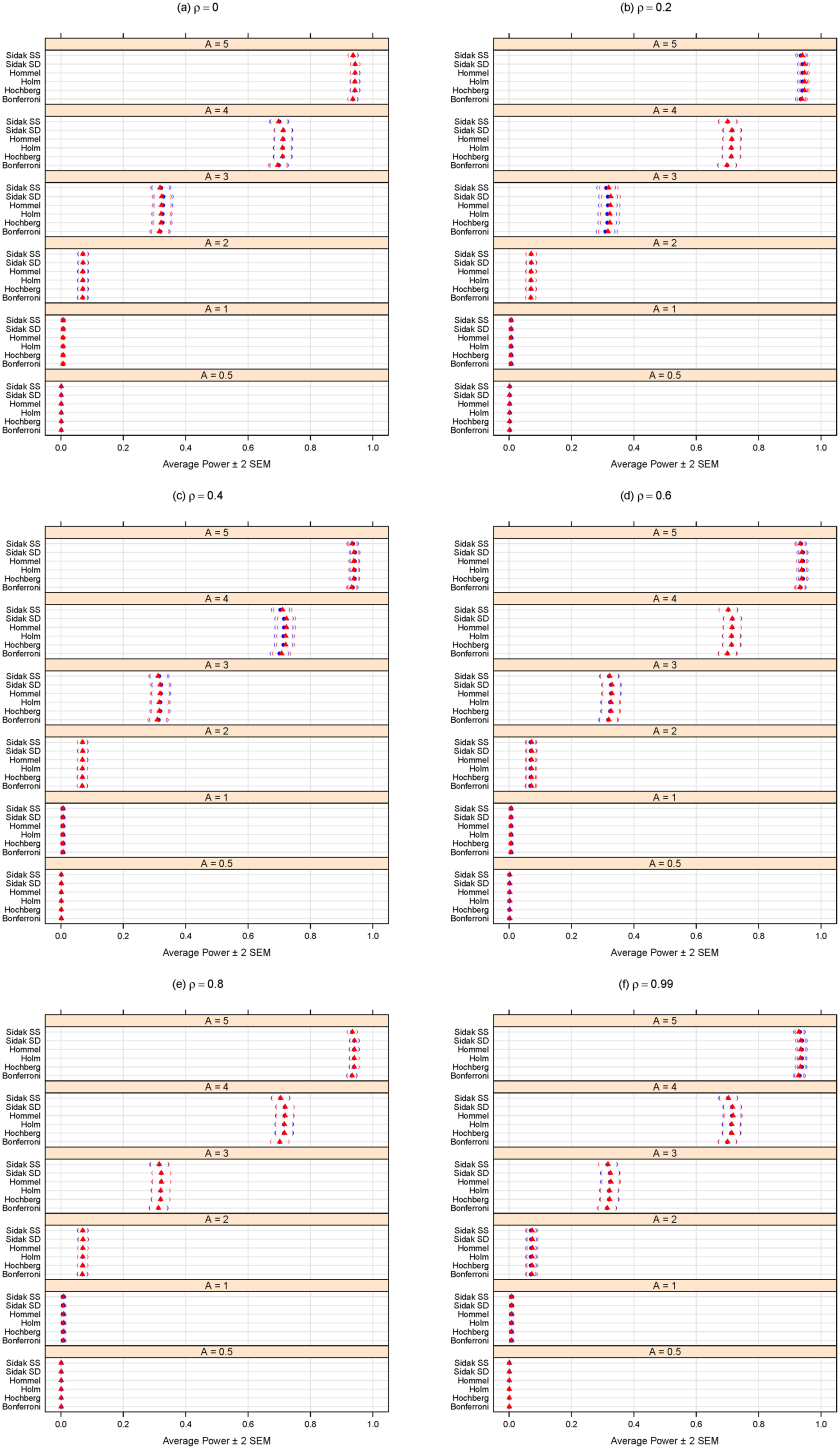
Average power for different methods purporting to control the FWER at *α* = 0.05. *A* can be thought of as the magnitude of differential abundance for truly differentially abundant features, and *ρ* is the true correlation within blocks of dependent tests. The blue solid circles represent the case of 18 dependent tests (out of 100 total), whereas the red solid triangles are for the case of 36 dependent tests. Parentheses indicate ± 2 SEM.

**Fig 3:**
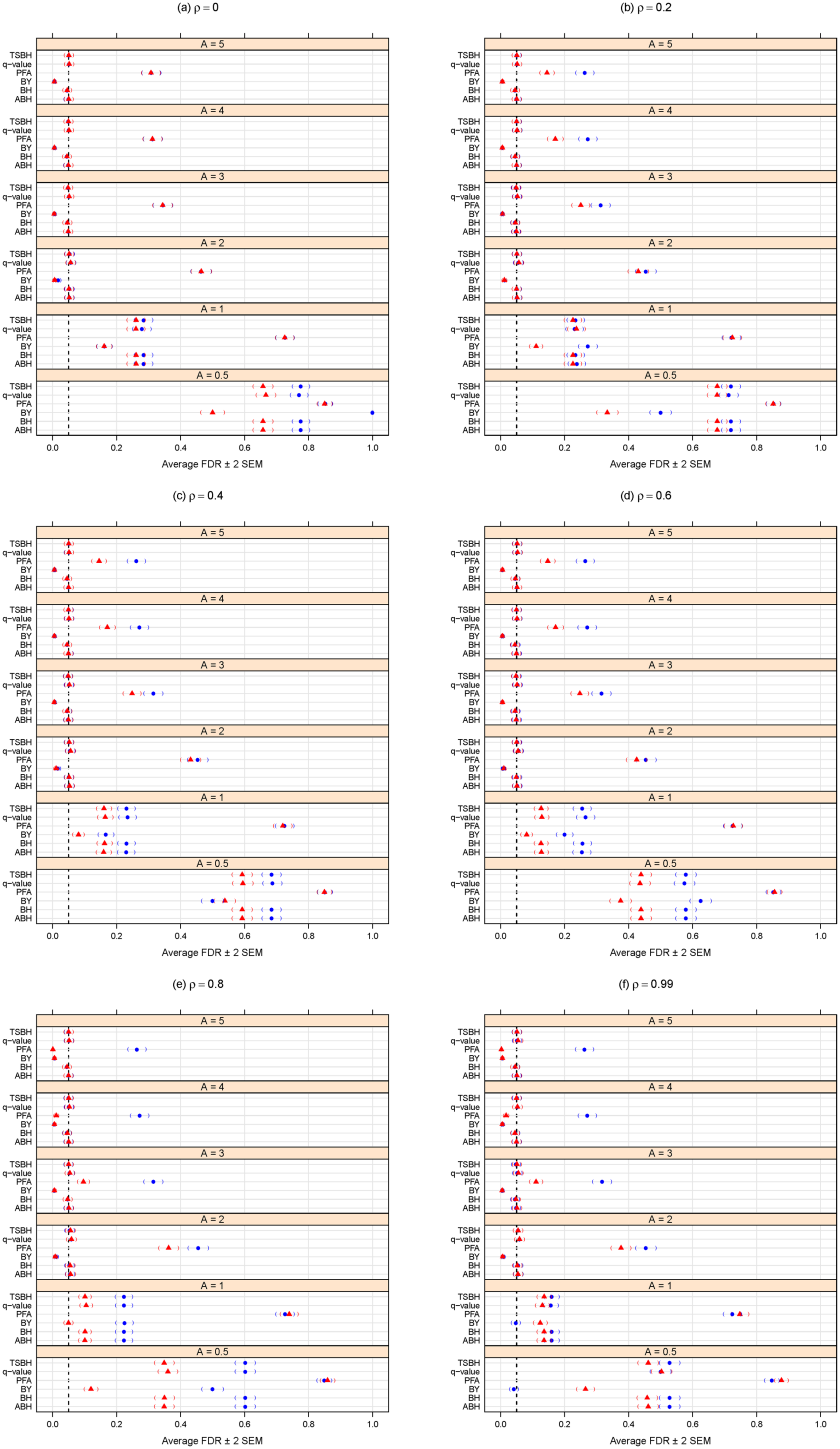
Average FDR for different methods purporting to control the FDR at *α* = 0.05. *A* can be thought of as the magnitude of differential abundance for truly differentially abundant features, and *ρ* is the true correlation within blocks of dependent tests. The blue solid circles represent the case of 120 dependent tests (out of 2000 total), whereas the red solid triangles are for the case of 360 dependent tests. Parentheses indicate ± 2 SEM.

**Fig 4:**
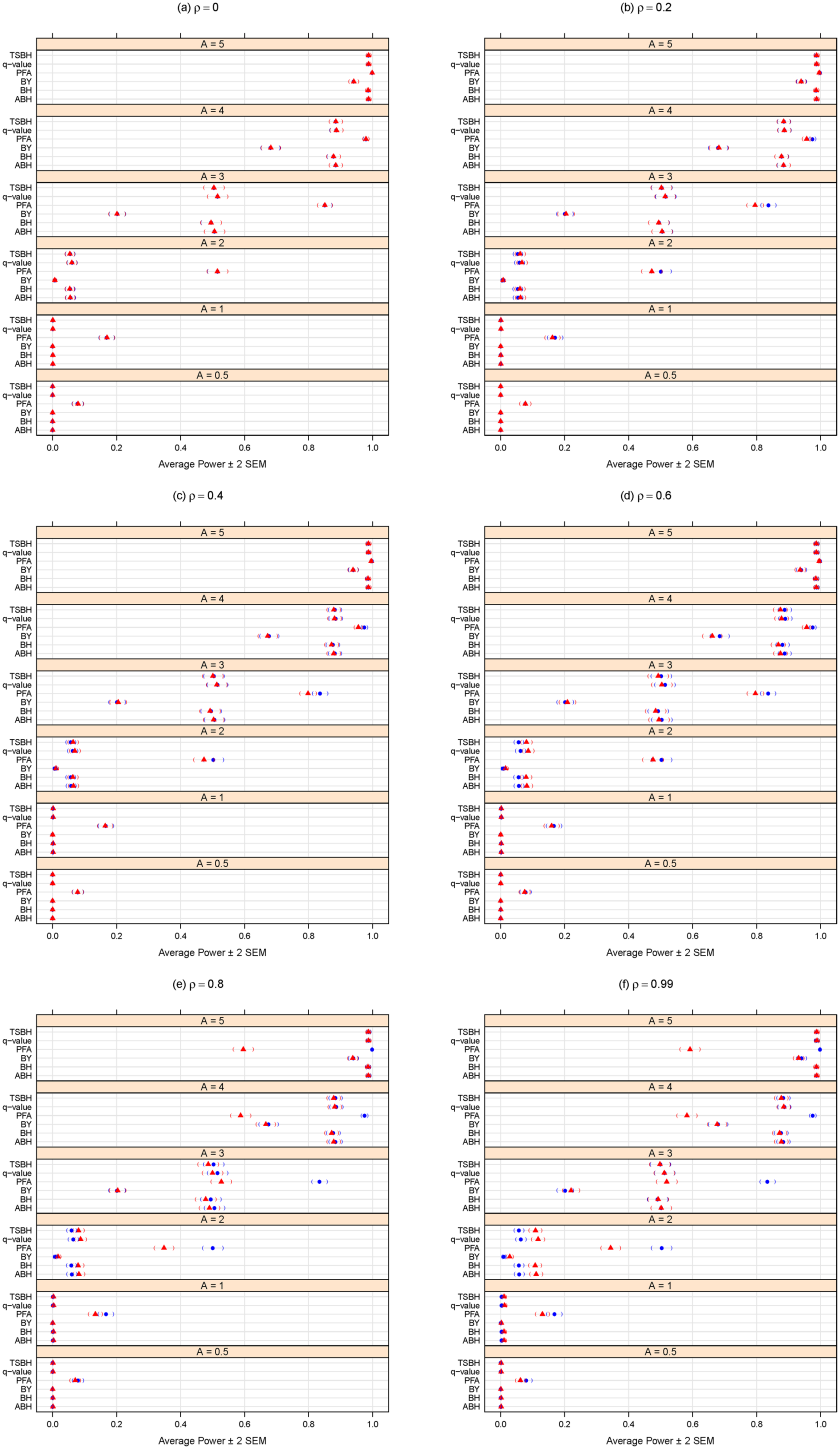
Average power for different methods purporting to control the FDR at *α* = 0.05. *A* can be thought of as the magnitude of differential abundance for truly differentially abundant features, and *ρ* is the true correlation within blocks of dependent tests. The blue solid circles represent the case of 120 dependent tests (out of 2000 total), whereas the red solid triangles are for the case of 360 dependent tests. Parentheses indicate ± 2 SEM.

In comparing the performance of the multiple testing adjustment methods considered in the “Methods: controlling the error rates” Section, it is important to consider the trade-off between specificity (one minus the type I error rate) and sensitivity (statistical power). A statistical method could achieve high power by automatically rejecting all null hypotheses, but this would negatively affect specificity. Conversely, adopting an overly-conservative approach that would reject hardly any (or even no) null hypotheses could maintain excellent specificity (i.e., a very low type I error rate rate) but would have poor statistical power. For this reason, the simulation results summarized here in Figs 1, 2, 3 and 4 consider both the average FDR (or FWER) control (as a form of specificity) and average statistical power (sensitivity). For convenience and clarity in visualization, the FDR (or FWER) and power are considered separately in Figs 1, 2, 3 and 4. For a simultaneous representation of the FDR (or FWER) and power results, see Section C of S1 File.

Figs 1 and 2 summarize the results of the simulation for the FWER-controlling methods. If the interest is to control the FWER, we note that all the FWER methods do indeed control the FWER equally well at the chosen *α* level (0.05 here), even in the presence of block-correlation positively-dependent tests, regardless of effect size (*A*), degree of dependence (*ρ*), or size of dependence group (Fig 1). As expected, Fig 2 shows these methods’ power increases for larger magnitudes of differential abundance (i.e., larger effect sizes *A*). Power does not appear to be affected by increasing levels of dependence (i.e., larger *ρ*) or dependence group size. Regardless of effect size (*A*) or degree of dependence (*ρ*), it appears best to use the Sidak SD, Hommel, Holm, or Hochberg methods, as there is a modest (but consistent) power loss in the Bonferroni and Sidak SS methods (Fig 2).

Figs 3 and 4 summarize simulation results for the FDR-controlling methods. FDR control (Fig 3) and statistical power (Fig 4) both improve, as expected, for larger magnitudes of differential abundance (i.e., larger effect sizes *A*.)

Fig 3 shows that increasing levels of dependence (i.e., larger *ρ*) appears to improve FDR control for tests of small effects (such as *A* = 0.5), but has no clear effect for larger *A*.

Fig 3 indicates that increasing the dependence group size (360 vs. 120) results in lower FDR when the effect is small (*A* = 0.5) and *ρ* is larger. Larger dependence group size also appears to result in a modest gain in (already poor) power (see Fig 4) among most FDR-controlling methods when the effect is moderate (such as *A* = 2) and *ρ* is larger. However, for larger *A* and larger *ρ*, Fig 4 shows a possible (if negligible) loss of power.

Of the methods purporting to control the FDR, the PFA method generally has the best power (Fig 4), but Fig 3 shows that, at least for this large number of tests and for the block-correlation positively-dependent covariance structure shown in Eq 1, the PFA method fails to provide even reasonable control of the FDR. We note that this PFA performance includes the best-case scenario of treating the covariance matrix as known. Additional simulations described in Section B of S1 File suggest that, at least for certain block-correlation positively-dependent covariance structures, the PFA method may provide better FDR control for smaller numbers of tests, but for larger numbers of tests (in the thousands), the PFA method does not provide the desired FDR control.

If the interest is to control the FDR, we note that, for at least moderate effect sizes (*A* ≥ 2), the FDR methods (other than PFA) do indeed control the FDR at the chosen *α* level (0.05 here), regardless of the degree of dependence (*ρ*) (Fig 1). The Benjamini and Yekutieli procedure (BY) gives the most conservative control of the FDR (Fig 1), but at a noticeable loss of power (Fig 2). Regardless of effect size (*A*) or degree of dependence (*ρ*), it appears best to use the two stage Benjamini and Hochberg procedure (TSBH), the q-value method, or the adaptive Benjamini and Hochberg procedure (ABH) to control the FDR, even when positive block correlation dependence is present.

We conclude with a few caveats. First, the multiple hypothesis testing literature is evolving, so the above recommendations will not necessarily remain the best in perpetuity. Also, we only considered a certain class of dependence among test results, and any simulation study can not reasonably consider all possible conditions (see the concluding two paragraphs of the “Methods: simulation analysis” Section above). Nevertheless, these results do provide a concrete comparison of multiplicity adjustment methods and give some insight as to the effects of degree of differential feature abundance (*A*), degree of dependence among tests (*ρ*), and sizes of dependence groups on error rate control and power. In addition, this comparison and all the panels of Figs 1–4 are completely reproducible using the R code provided in S2 File.

## Supporting information

S1 File Background information on previous literature, including multiple testing adjustment methods and dependence among test results. (PDF)

S2 File R code to reproduce the entire simulation, including data analysis and summary figure panels. (R)
