# Supplementary material for: A comparison of multiple testing adjustment methods with block-correlation positively-dependent tests
Source: PLoS One. 2017 Apr 28;12(4):e0176124. doi: 10.1371/journal.pone.0176124 (PMC5409054; doi:10.1371/journal.pone.0176124)
Supplement: S1 File — (PDF) [file pone.0176124.s001.pdf]

Background and Supplemental Material (S1 File) for "A Comparison of Multiple Testing Adjustment Methods with Block-Correlation Positively-Dependent Tests"

A. Methods: controlling the error rates

Suppose a study involves testing simultaneously m null hypotheses H_k, $k = 1, 2, ..., m$. In a general context, each H_k represents testing the differential abundance of a feature k. Let m_0 be the (unknown) number of true null hypotheses. This m_0, along with some random variables (S, T, U, and V) of m specific hypotheses are summarized in Table 1, based on Benjamini and Hochberg (1995) [1]. In this Table, R is the total number of significant (rejected) tests (i.e., R is the total number of declared significant features). The typical objective is to control the number of false positives V (type I errors), while minimizing the number of false negatives T (type II errors) in multiple hypothesis testing to achieve higher statistical power.

Table 1. Symbolic representation of numbers of errors that could occur when simultaneously testing m hypotheses

	Declared tests		
Hypotheses	Non-significant	Significant	Total
Null	U	V	m_0
Alternative	T	S	m-m_0
Total	m-R	R	m

Generally, there are two types of multiple testing methods: those that control the FWER, and those that control the FDR. Methods involved in controlling the FWER are discussed in Section , and methods involved in controlling the FDR in Section . Throughout Sections and , $p_1, p_2, ..., p_m$ are p-values of a set of given null hypotheses $H_1, H_2, \ldots, H_m$ (and with corresponding test statistics X_1, ..., X_m); some methods involve the ordered p-values $p_{(1)} \leq p_{(2)} \leq \ldots \leq p_{(m)}$, with ordered p-value $p_{(i)}$ corresponding to null hypothesis $H_{(i)}$ and test statistic $X_{(i)}$.

Methods involved in controlling the family wise error rate (FWER)

The FWER is defined as the probability of one or more false positive events (or type I errors) among all the hypotheses when conducting multiple hypothesis tests. That is,

$$FWER = P[V > 0]. \tag{1}$$

Bonferroni procedure

In multiple comparisons, the Bonferroni correction [2] is widely used to control the probability that a true null hypothesis is incorrectly rejected, although it gives a conservative upper bound on the FWER. The Bonferroni procedure rejects hypothesis

H_i when $p_i \leq \{\alpha/m\}$, using the Bonferroni inequality [2,3]. For any combination of true and false null hypotheses, this procedure ensures that the probability of any type I error does not exceed the desired level α; that is, this Bonferroni procedure provides strong control of the FWER.

Šidák's single step (SS) and step-down (SD) procedures

Šidák (1967) [4] proposed an alternative method of multiple comparison for controlling the FWER at level α. The main motivation was a criticism of overly-conservative results from the Bonferroni correction. Considering the Bonferroni-adjusted significance threshold α/m, it is obvious that we compare the p-values to a very small significance level when there is a large number of hypotheses. So, in order to improve the conservative result of multiple comparisons, the Šidák Single Step (SS) procedure [3] rejects null hypothesis H_i when $p_i \leq 1-(1-\alpha)^{1/m}$, for $i = 1, 2, \ldots, m$. The SS procedure is more powerful than the Bonferroni correction, but the gain of SS is small [5], and the SS method assumes independence while the Bonferroni approach controls the FWER even with arbitrary dependence among tests. Both the Bonferroni and the SS procedures do not require ordering the p-values prior to adjustment.

The step-down (SD) idea is briefly described here. If ordered p-value $p_{(i)} \geq \alpha$, then no hypotheses $H_{(j)}$ for $j > i$ will be rejected, and we can step down from adjusting for m tests to adjusting for $m-i+1$ tests. Holland and Copenhaver (1987) [5] used *Šidák*'s SS procedure, utilizing the SD idea, to gain in statistical power over the Bonferroni method. We call this test *Šidák*'s SD procedure. The SD procedure rejects any null $H_{(i)}$ if $p_{(i)} \leq 1-(1-\alpha)^{1/(m-j+1)}$ for any $j = 1, 2, \ldots, i$ [3,5].

Holm procedure

Holm (1979) [6] demonstrated a new procedure of multiple comparisons that controls the type I error rate at level α. Regardless of various combinations of true and false null hypotheses, this procedure considers significance tests sequentially (i.e "one at a time") as long as the previous result rejects. It has been called "the sequentially rejective Bonferroni test" because at each step it uses a Bonferroni correction over the remaining tests to be considered.

The Holm procedure can be described using the ordered p-values. One can execute the Holm step-down procedure for a given α level as follows:

Step 1: Reject $H_{(1)}$ if $p_{(1)} \leq \alpha/m$

Step 2: Reject $H_{(2)}$ if $p_{(2)} \leq \alpha/(m-1)$

...

Step m: Reject $H_{(m)}$ if $p_{(m)} \leq \alpha$.

Generally, the Holm method rejects hypothesis $H_{(i)}$ when $p_{(i)} \leq \alpha/(m-i+1)$ for $i = 1, 2, \ldots, m$. The procedure stops at the smallest i, where the p-value $p_{(i)}$ exceeds the given level test. Then, only those hypotheses already tested are rejected. This procedure gains more power than the Bonferroni method, but the actual power gain is not large [6]. Since the Holm procedure is a closed testing procedure, it controls the FWER for all the m hypotheses at level α in the strong sense (meaning for any combination of true and false null hypotheses).

The closed testing procedure proposed by Marcus, Peritz and Gabriel (1976) [7] is described as follows. Suppose there are m hypotheses $H_1, H_2, ..., H_m$ and the desired overall type I error rate is α. If not only any of these elementary hypotheses, say H_k ($k \in \{1, 2, \ldots, m\}$), is significant, but also all of the intersection hypotheses that include

H_k are significant, then H_k will be declared significant based on the closed testing principle.

It is worth noting that while Holm (1979) [6] mentioned the desirability of independent test statistics from the m null hypotheses ("not for computational reasons but because a good experimental design requires the different hypotheses to be tested by variables 'not related to each others' "), the Holm procedure itself does not require independence of test statistics.

Hommel procedure

Hommel (1988) [8] presented a multiple-testing approach where inference for $H_{(i)}$ depends on an index j, where $j = \max\{i \in \{1, 2, ..., m\} \colon p_{(m-i+k)} > (k\alpha/i); k \in \{1, 2, ..., i\}\}$. In this approach, $H_{(i)}$ is declared significant when $p_{(i)} \leq \{\alpha/j\}$. Otherwise, reject all $H_{(i)}$ if j does not exist. In general, if test statistics are independent, then the Hommel (1988) [8] method controls the FWER at level α, although it does work for some dependence structures among test results [8].

Hochberg procedure

The Hochberg procedure [9] applies the step-up technique in which one rejects all $H_{(i')}$ with $p_{(i)} \leq \alpha/(m-i+1)$ for $(i^{'} \leq i)$, where $i = m, m-1, \ldots, 1$. At the initial stage, if $p_{(m)} \leq \alpha$, then one rejects all $H_{(i')}$ $(i^{'} = m, m-1, ..., 1)$; otherwise, one accepts $H_{(m)}$. Then the procedure rejects the rest of the hypotheses, $H_{(i')}$ $(i^{'} = m-1, m-2, \ldots, 1)$ with $p_{(m-1)} \leq \{\alpha/2\}$, and so on.

Like the Hommel procedure (see Section), this procedure also performs well on independent test statistics. But the Hommel procedure increases the power of the tests while controlling the FWER. Sarkar (1998) [10] showed that the Hochberg procedure controls the FWER when test statistics follow the multivariate total positivity of order 2 (that is, MTP_2) dependence condition (see Section).

Methods controlling the false discovery rate (FDR)

Recalling Table 1 and according to Benjamini and Hochberg (1995) [1], the False Discovery Proportion (FDP) is the number of false positives (or false rejections) divided by the number of rejections. That is,

$$Q = \frac{V}{max(R,1)} = \begin{cases} \frac{V}{R}, & \text{if } R > 0 \\ 0, & \text{otherwise} \end{cases} \tag{2}$$

where Q is the false discovery proportion. The FDR is defined as the expected FDP. That is,

$$FDR = E(Q). \tag{3}$$

Benjamini and Hochberg (BH) procedure

Benjamini and Hochberg (1995) [1] provided a completely different idea of controlling error rates in multiple hypothesis testing. The procedure involves a step-up technique for non-parametric statistics in order to control the error rates. They suggested the multiplicity problem will be reduced if we only consider the FDP instead of the usual idea of the probability of making at least one type I error among all tests. In addition, the exact controlling will be accomplished by the FDR while providing a smaller type II error rate.

Benjamini and Hochberg proposed a linear step-up technique, considering the Simes (1986) [11] test, that ensures the FDP converges to a desired level α in the 1st order mean. Their proposed method, referred to as the BH procedure, also provides a non-parametric and finite sample method for choosing the p-value threshold, which is often more powerful than the traditional methods associated with controlling the FWER.

The linear step-up BH procedure rejects all hypotheses $H_{(k)}$ with $k \leq \max \{i : p_{(i)} \leq (i\alpha/m)\}$. Otherwise, the process stops when k does not exist. For independent tests the BH procedure controls the FDR at level α.

Following Table 1 and Eq 3, one of the important features of the FDR mentioned is that it will not be controlled at level α if $m_0 = m$, even if $Q = 1$ [1]. Moreover, controlling $(V/R|R > 0)$ (see Eq 2) is not possible when $Q = 1$. Thus, the alternative formulation of the FDR is

$$P(R > 0)E(V/R|R > 0). \tag{4}$$

Benjamini and Yekutieli (BY) procedure

Benjamini and Yekutieli (2001) [12] extended the work of the original BH procedure (see Section) for dependent test statistics (or dependent p-values). They pointed out that the BH procedure controls the FDR at level $\frac{m_0}{m}\alpha \leq \alpha$ when the test statistics have a positive regression dependence structure (see Section). However, when the test statistics are negatively correlated or there is a different dependency, a modified BH procedure, referred to here as the BY procedure, is needed to control the FDR at the same level $\frac{m_0}{m}\alpha \leq \alpha$.

This BY method multiplies the BH-adjusted p-values by $\sum_{i=1}^{m} \frac{1}{i}$, rounded down to (or truncated at) 1 as necessary. Equivalently, the BY method replaces α in the BH method with $\alpha / \sum_{i=1}^{m} \frac{1}{i}$.

Adaptive Benjamini and Hochberg (ABH) procedure

Benjamini and Hochberg (2000) [13] modified the original BH procedure (see Section) by estimating m_0 (i.e., the number of true null hypotheses) in Table 1. The original BH procedure does not require estimation of m_0, while this procedure depends on the knowledge of m_0. Assuming independent tests to estimate m_0 by the original BH procedure, the adaptive BH procedure, referred to here as the ABH procedure, involves the following steps [14]:

Step 1: Use the linear step-up procedure (that is, original BH procedure) at α, and if no hypothesis is rejected stop; otherwise, proceed.

Step 2: Estimate $m_0(k)$ by $(m + 1 - k)/(1 - p_{(k)})$, where $k = \max\{i\colon p_{(i)} \leq (i\alpha/m)\}$ for which we reject all $H_{(i)}$ $(i = 1, 2, \ldots, k)$.

Step 3: Starting with $k = 2$ stop when for the first time $m_0(k) > m_0(k - 1)$.

Step 4: Estimate $\hat{m}_0 = \min\{m_0(k), m\}$ rounding up to the next highest integer.

Step 5: Use the linear step-up procedure with $\alpha^{'} = m\alpha/\hat{m}_0$.

The ABH procedure controls the FDR exactly at level α, while the original BH procedure was shown to provide a slightly conservative upper bound. In addition, the ABH procedure has greater power than the original BH procedure [13, 14].

Two stage Benjamini and Hochberg (TSBH) procedure

According to Benjamini et al. (2006) [14], the two-stage linear step-up procedure of the BH procedure (see Section), which is referred to here as the TSBH procedure, is summarized in the following way:

Step 1: Use the linear step-up procedure (or the original BH procedure) at level $\alpha^{'} = \alpha/(1+\alpha)$. Let r_1 be the number of rejected hypotheses. If $r_1 = 0$ do not reject any hypothesis and stop; also if $r_1 = m$ reject all m hypotheses and stop; otherwise proceed.

Step 2: Assume $\hat{m}_0 = m - r_1$.

Step 3: Use again the linear step-up procedure with $\alpha^{''} = m\alpha^{'}/\hat{m}_0$.

In Step 2, the TSBH procedure involves estimating m_0 (see Table 1) with the constraint

$$m_0 \leq m - (R - V). \tag{5}$$

The BH procedure (see Section) is used in the initial stage, ensuring $E(V/R) \leq \{m\alpha/m_0\}$, so that $V \leq \{\alpha m_0 R/m\}$. Substituting V into Eq 5, we obtain the following relations:

$$m_0 \leq m - \left(R - \frac{\alpha m_0 R}{m}\right) \tag{6}$$

and

$$m_0 \leq \frac{(m-R)}{1-\frac{R\alpha}{m}} \leq \frac{(m-R)}{(1-\alpha)} \leq (m-R)(1+\alpha). \tag{7}$$

The right-most bound of Eq 7 is inherently used in the TSBH procedure. This procedure also controls the FDR at level α when test statistics are independent. Benjamini et al. (2006) [14] showed that the TSBH procedure controls the FDR below but close to the nominal level α and provides higher power than the original BH procedure when tests are correlated.

q-value method

In the original BH procedure (see Section), the rejection of all hypotheses H_i $(i = 1, 2, \ldots, k)$ relies on $k = max\{i\colon p_{(i)} \leq i\alpha/m\}$. However, the estimation process of the maximum number of rejected hypotheses (i.e., "$\hat{k}$") from each possible combination of hypotheses is not clearly explained in the BH procedure [15]. Storey (2002) [15] also pointed out that the BH procedure may not give a reliable estimate for $\hat{k}$ when a large number of hypotheses are considered, and hence it affects controlling the FDR at the level α "for all values of m_0 (i.e., the number of true null hypotheses) simultaneously".

To address the weaknesses of the BH procedure, Storey (2002) [15] demonstrated a new way of controlling the error rates with the positive False Discovery Rate (pFDR) at the desired level α. The error rate, pFDR, preserves information about m_0 by applying point estimation techniques in a Bayesian framework. Storey (2002) [15] introduced a new term "q-value" that quantifies the pFDR. He defined the pFDR as in Eq 8:

$$pFDR = E(V/R|R > 0). \tag{8}$$

That is, the pFDR is the expected portion of erroneously rejected hypotheses among all rejected hypotheses when significant findings have occurred. Where the BH approach

uses threshold $k = max\{i\colon p_{(i)} \leq i\alpha/m\}$, and rejects all hypotheses H_i $(i = 1, 2, \ldots, k)$, Storey(2002) [15] proposed to use a fixed threshold t (for significance of p-values) and estimate the resulting FDR; the choice of t is such that the estimated FDR is less than or equal to α.

Storey (2002) [15] identified the q-value as analogous to the p-value; the q-value measures the error rate with respect to the pFDR, but the p-value measures the error rate with respect to the type I error.

Principal factor approximation (PFA) method

Fan *et al.* (2012) [16] presented an alternative approach to estimate the FDR, similar to the q-value framework (see Section), but paying particular attention to the potential arbitrary dependence among test statistics. This Principal Factor Approximation (PFA) approach treats test statistics $X_1, \ldots, X_m$ as normally distributed with mean vector $(\mu_1, \ldots, \mu_m)$ and covariance matrix $\underset{\sim}{\Sigma}$. The basic idea of this PFA approach "is to first take out the principal factors that derive the strong dependence among observed data" X_1, ..., X_m "and to account for such dependence in calculation of the false discovery proportion" [16]. In our implementation of this method, we treated the $\underset{\sim}{\Sigma}$ covariance matrix as known, and used stated default parameters in the pfa.test function of the R package pfa [17].

Dependence among test results: PRDS and MTP$_2$

The test statistics $\underset{\sim}{X} = (X_{(1)}, \ldots, X_{(m)})$ corresponding to p-values $p_{(1)} \leq \ldots \leq p_{(m)}$ (and null hypotheses $H_{(1)}, \ldots, H_{(m)}$) are sometimes assumed independent by multiplicity correction methods, but in many contexts (as in the genomic, spatial epidemiology, and brain imaging studies mentioned in the main article), this assumption is suspect. Benjamini and Yekutieli (2001) [12] define different dependence types among test statistics. While those authors provide technical definitions, we briefly summarize here the main dependence types.

Let $I_0 \subseteq \underset{\sim}{X}$ be the set of test statistics corresponding to true null hypotheses. The dependence type emphasized by Benjamini and Yekutieli (2001) [12] is positive regression dependency on each one from a subset I_0, or PRDS. This can be interpreted as saying that $H_{(1)}$ is least likely to be in I_0, followed by $H_{(2)}$, and so on, until $H_{(m)}$ is the most likely to be in I_0. Benjamini and Yekutieli (2001) [12] showed that, when the joint distribution of $\underset{\sim}{X}$ is PRDS, the original BH procedure controls the FDR at level $\frac{m_0}{m}\alpha \leq \alpha$. They also showed that, for any joint distribution of $\underset{\sim}{X}$ (even if not PRDS), their BY procedure controls the FDR at level $\frac{m_0}{m}\alpha \leq \alpha$.

A special case of PRDS (which is easier to show than PRDS [12]) is multivariate total positivity of order 2, or MTP$_2$. In this dependence type, let $f_X(x)$ be the density function of $\underset{\sim}{X}$. Then $\underset{\sim}{X}$ (and its density) are MTP$_2$ if for all length-m vectors $\underset{\sim}{x}$ and $\underset{\sim}{y}$,

$$f_X(\underset{\sim}{x}) \cdot f_X(\underset{\sim}{y}) \leq f_X(min(\underset{\sim}{x}, \underset{\sim}{y})) \cdot f_X(max(\underset{\sim}{x}, \underset{\sim}{y})) \tag{9}$$

where the min and max are evaluated componentwise [12].

According to Nichols and Hayasaka (2003) [18], "Gaussian data with positive correlations will satisfy the PRDS condition," a fact that we use in the simulation section of the article to compare performance of the aforementioned multiplicity adjustment methods when test results are not independent.

Abbreviations of relevant procedures

Abbreviations of the multiple comparison procedures discussed to this point are shown with their corresponding Section numbers in Table 2. These procedures are used to adjust p-values in our simulation analysis and to visualize results in the article.

Table 2. Abbreviations of multiple comparison procedures, used in the text (and summarized in given Section numbers).

Procedures	Abbreviation	Section
Bonferroni procedure	Bonferroni	
Šidák single step procedure	Sidak SS	
Šidák step down procedure	Sidak SD	
Holm procedure	Holm	
Hommel procedure	Hommel	
Hochberg procedure	Hochberg	
Benjamini and Hochberg procedure	BH	
Benjamini and Yekutieli procedure	BY	
Adaptive Benjamini and Hochberg procedure	ABH	
Two stage Benjamini and Hochberg procedure	TSBH	
q-value method	q-value	
Principal factor approximation	PFA	

B. Additional simulations for PFA

Based on the FDR control (or lack thereof) exhibited by the PFA method in Fig. 3 of the article, we considered an additional simulation to examine if there were conditions in which the PFA method would provide FDR control. It is beyond the scope of our work to exhaustively characterize the performance of the PFA method; instead, we only consider if the PFA method can be shown to provide reasonable control of the FDR in some simulation settings.

We use a simulation set-up similar to that described in Section 3 of the article, including reference to μ, A, and ρ. Let m be the total number of tests (features); we consider m values ranging from 80 to 2000, and 10 percent of the tests have false null hypotheses. We assume six blocks of correlated tests, with constant within-block correlation ρ, and each block is comprised of 2.5 percent of the m tests. Three blocks correspond to false null hypotheses (using $A = 2$), and three blocks correspond to true null hypotheses ($A = 0$). Of the independent tests, most (82.5 percent of the m tests) correspond to true null hypotheses, but some (the remaining 2.5 percent of the m tests) correspond to false null hypotheses (again with $A = 2$). Across 100 simulations, we calculate the average and standard deviation of the FDR (when aiming for purported FDR control at level $\alpha = 0.05$), with results summarized (and showing approximate 95 percent confidence intervals) in Fig. S1 below.

Based on Fig. S1 below, it does appear that the PFA method provides better FDR control for lower numbers of tests, but does not appear to control the FDR at the stated α level. It should be noted that this simulation (which, along with the panels of Fig. S1, can be reproduced using R code found in S2 file) treats the $\underset{\sim}{\Sigma}$ covariance matrix as known, and uses stated default parameters in the pfa.test function of the R package pfa [17].

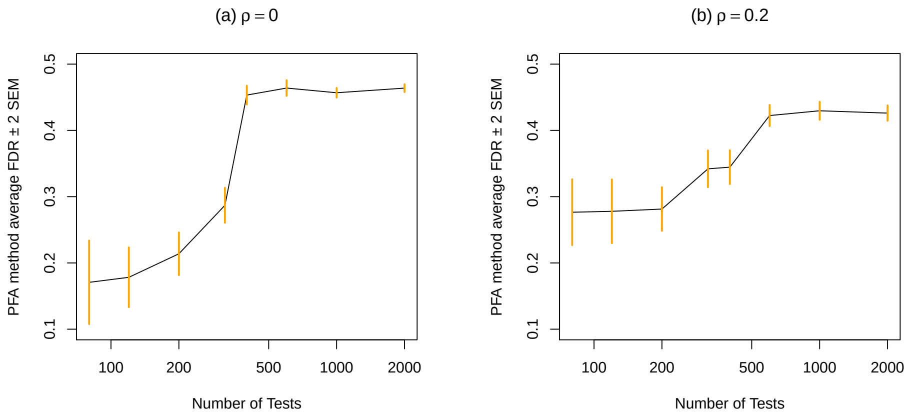

Fig. S1 Average PFA method FDR results across 100 simulations at various numbers of tests, with **(a)** independent ($\rho = 0$) and **(b)** positively-dependent ($\rho = 0.2$) tests. Vertical orange bars indicate $\pm$ 2 SEM. Horizontal axis tick marks are on the log scale.

C. Additional perspective on FDR/FWER vs. power

During the review process of this manuscript, a reviewer requested a simultaneous representation of the FDR (or FWER) and power results, to facilitate a comparison of their relative tradeoff. Accordingly, we present the following Figures S1a, S1b, and S1c:

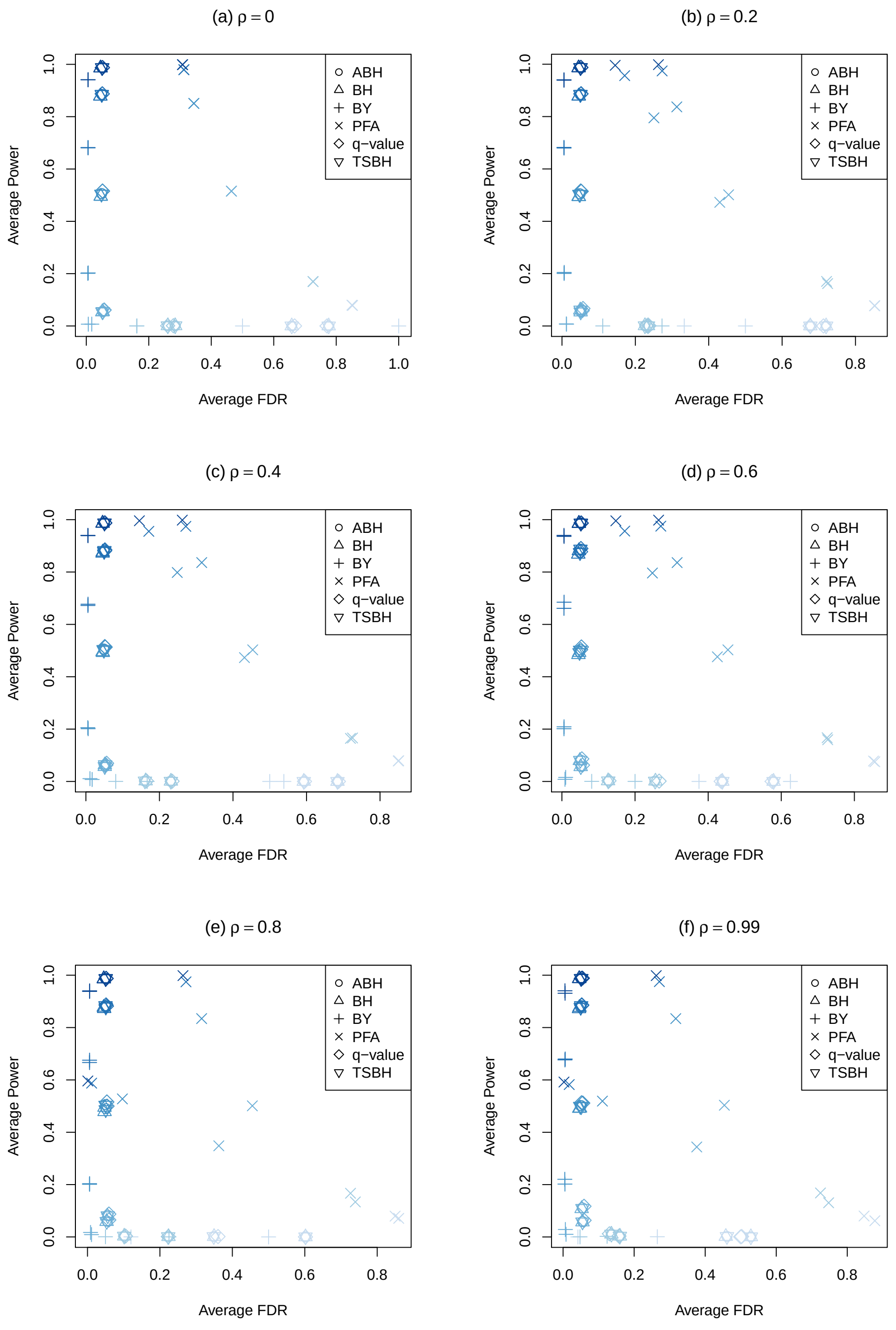

Fig. S1a Alternative simultaneous representation of the simulation results from Figs. 3 (FDR) and 4 (Power) from the main manuscript. Darker colors correspond to larger values of A, the magnitude of differential abundance.

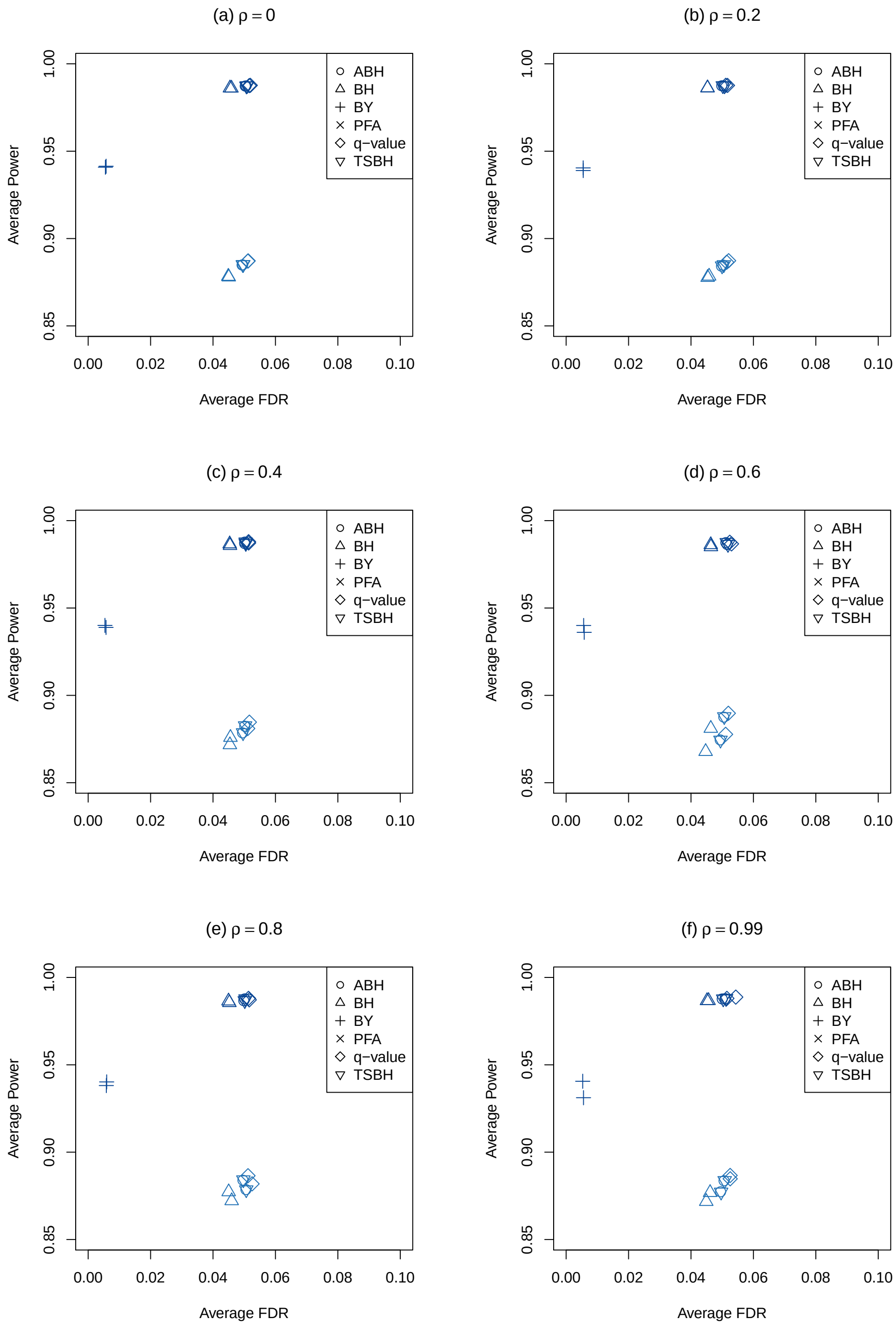

Fig. S1b Zoom-in of a portion of Figure S1a.

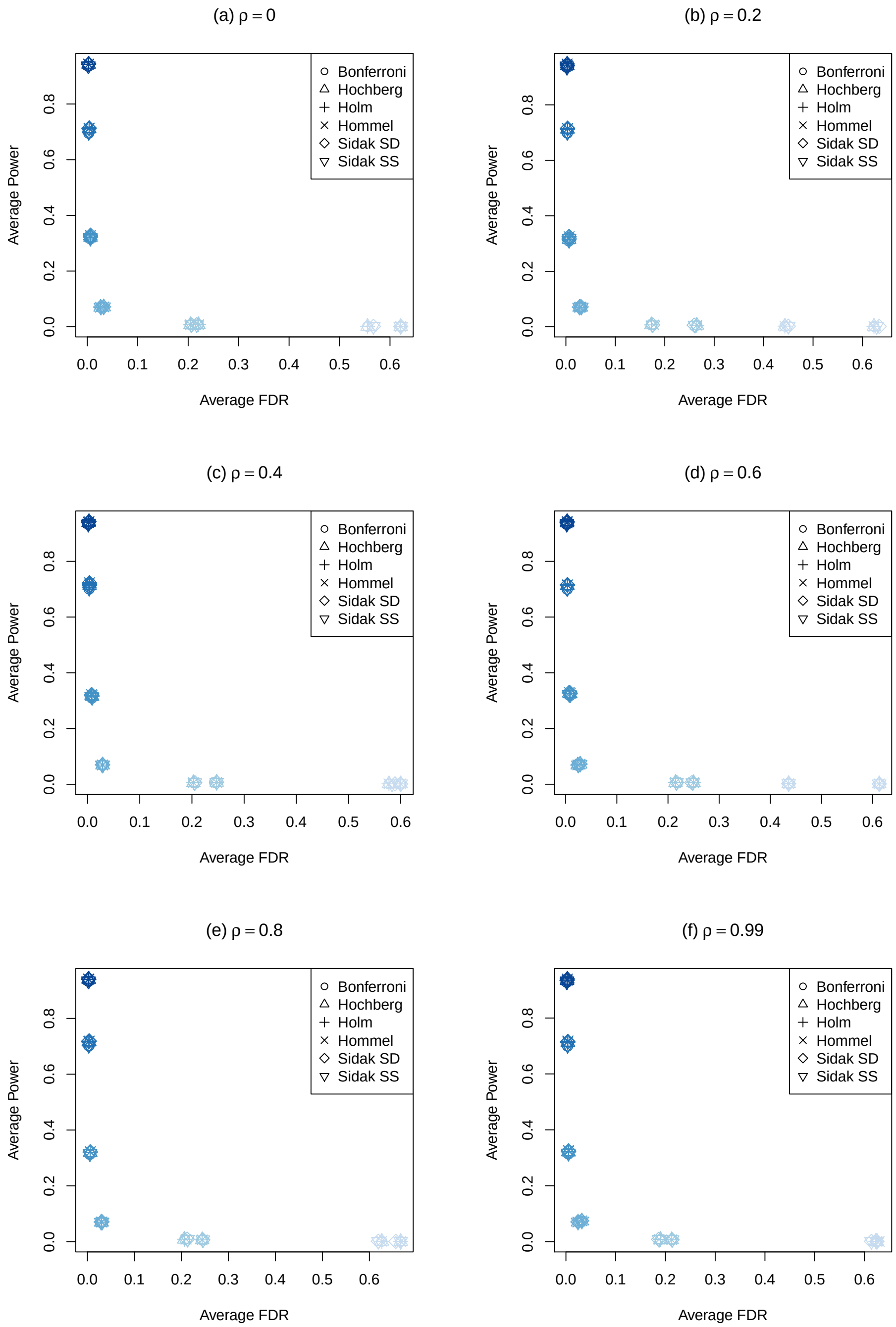

Fig. S1c Alternative simultaneous representation of the simulation results from Figs. 1 (FWER) and 2 (Power) from the main manuscript. Darker colors correspond to larger values of A, the magnitude of differential abundance.

Based on Figures S1a - S1c, as the magnitude of differential abundance (A) increases (i.e., as the plotting characters have a darker color), there is a general trend that the FDR and FWER decrease while the power increases. The shape of this relationship is quite different for the PFA method in Fig. S1a.

References

1. Benjamini Y, Hochberg Y. Controlling the false discovery rate: a practical and powerful approach to multiple testing. Journal of the Royal Statistical Society Series B (Methodological). 1995; p. 289–300.

2. Bonferroni CE. Teoria statistica delle classi e calcolo delle probabilita. Libreria Internazionale Seeber; 1936.

3. Dudoit S, Van Der Laan MJ. Multiple testing procedures with applications to genomics. Springer Science & Business Media; 2007. Available from: http://www.springerlink.com/content/978-0-387-49316-9.

4. Šidák Z. Rectangular confidence regions for the means of multivariate normal distributions. Journal of the American Statistical Association. 1967;62(318):626–633.

5. Holland BS, Copenhaver MD. An improved sequentially rejective Bonferroni test procedure. Biometrics. 1987; p. 417–423.

6. Holm S. A simple sequentially rejective multiple test procedure. Scandinavian Journal of Statistics. 1979; p. 65–70.

7. Marcus R, Eric P, Gabriel KR. On closed testing procedures with special reference to ordered analysis of variance. Biometrika. 1976;63(3):655–660.

8. Hommel G. A stagewise rejective multiple test procedure based on a modified Bonferroni test. Biometrika. 1988;75(2):383–386.

9. Hochberg Y. A sharper Bonferroni procedure for multiple tests of significance. Biometrika. 1988;75(4):800–802.

10. Sarkar SK. Some probability inequalities for ordered MTP2 random variables: a proof of the Simes conjecture. Annals of Statistics. 1998; p. 494–504.

11. Simes RJ. An improved Bonferroni procedure for multiple tests of significance. Biometrika. 1986;73(3):751–754.

12. Benjamini Y, Yekutieli D. The control of the false discovery rate in multiple testing under dependency. Annals of Statistics. 2001; p. 1165–1188.

13. Benjamini Y, Hochberg Y. On the adaptive control of the false discovery rate in multiple testing with independent statistics. Journal of Educational and Behavioral Statistics. 2000;25(1):60–83.

14. Benjamini Y, Krieger AM, Yekutieli D. Adaptive linear step-up procedures that control the false discovery rate. Biometrika. 2006;93(3):491–507.

15. Storey JD. A direct approach to false discovery rates. Journal of the Royal Statistical Society: Series B (Statistical Methodology). 2002;64(3):479–498.

16. Fan J, Han X, Gu W. Estimating false discovery proportion under arbitrary covariance dependence. Journal of the American Statistical Association. 2012;107(499):1019–1035.

17. Fan J, Ke T, Li S, Xia L. pfa: Estimates False Discovery Proportion Under Arbitrary Covariance Dependence; 2016. Available from: https://CRAN.R-project.org/package=pfa.

18. Nichols T, Hayasaka S. Controlling the familywise error rate in functional neuroimaging: a comparative review. Statistical Methods in Medical Research. 2003;12:419–446.
